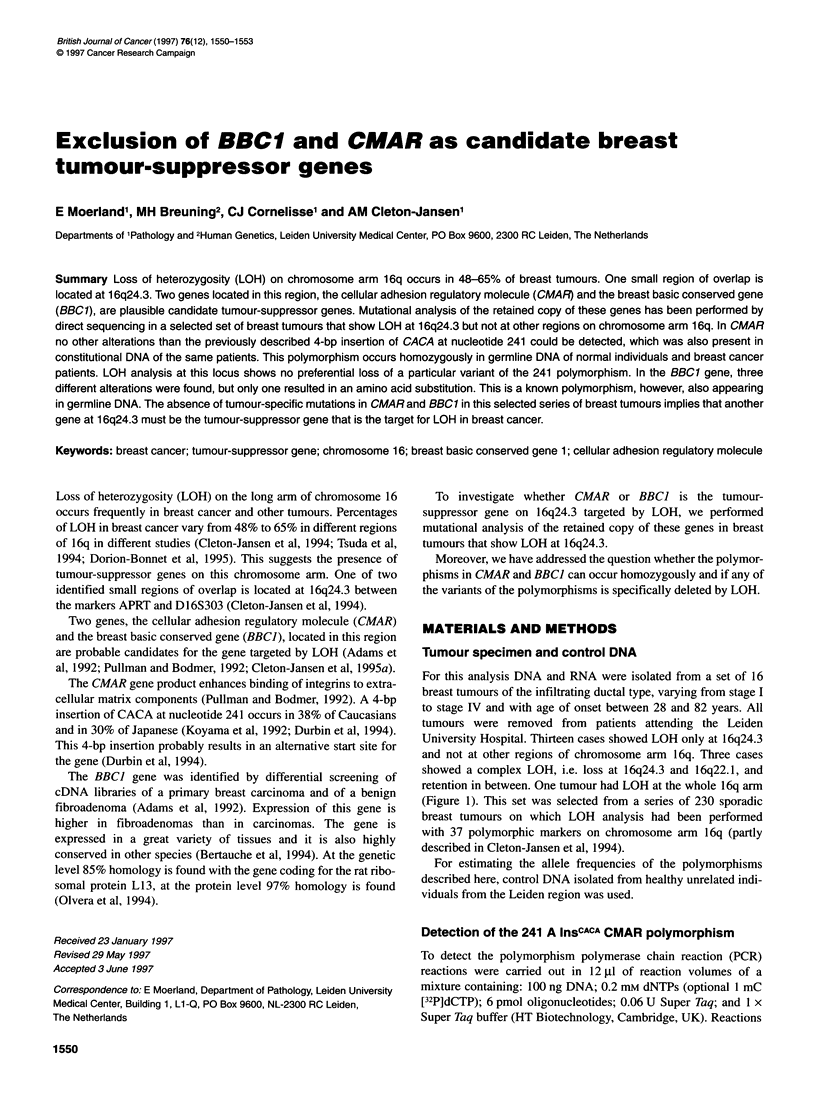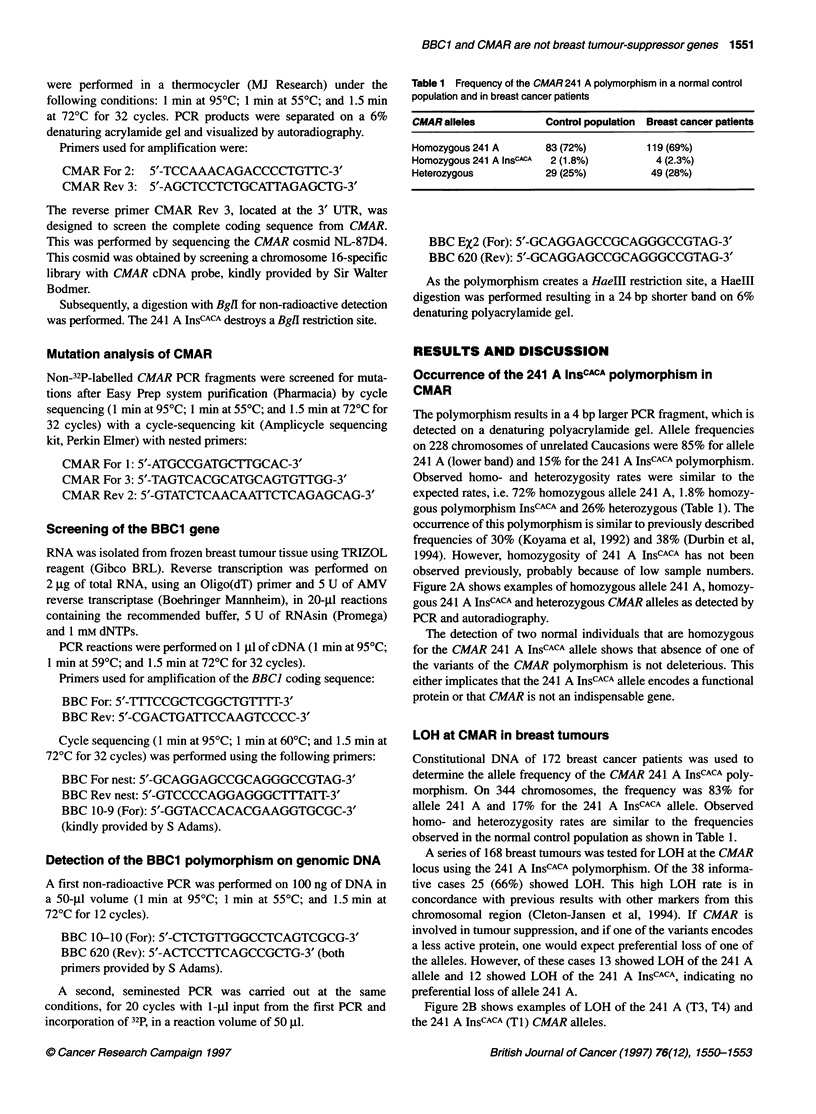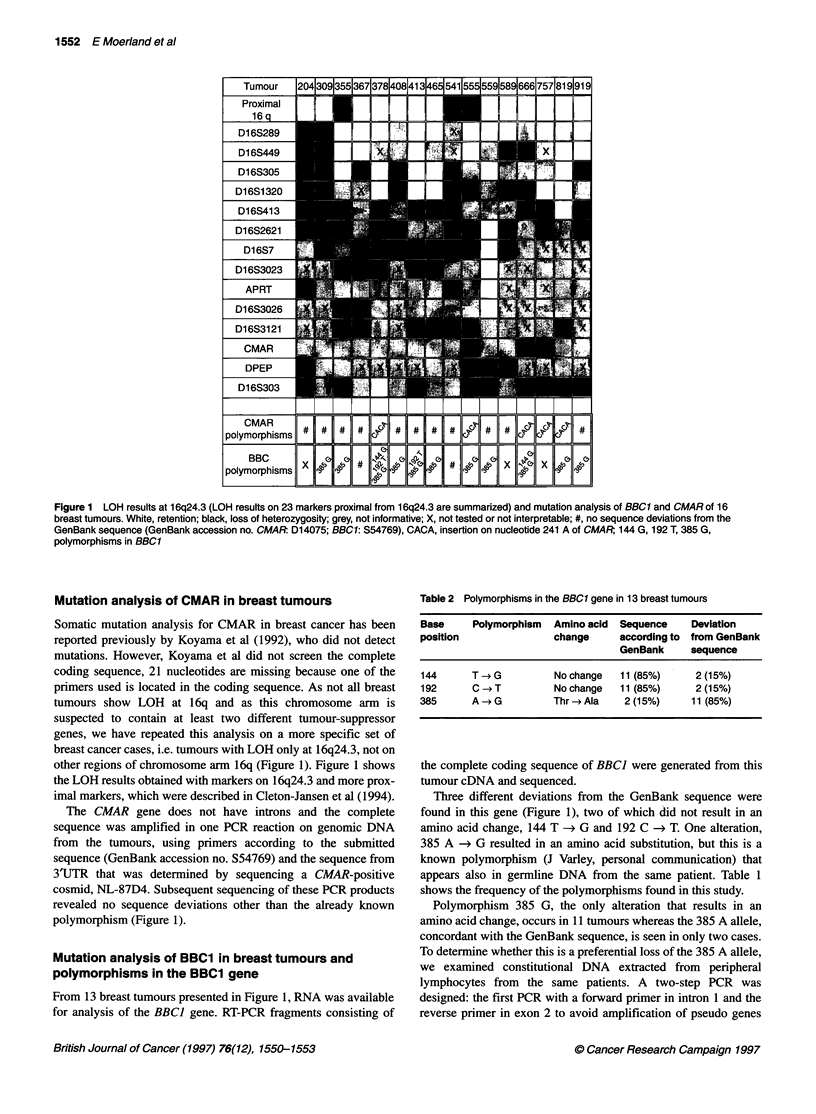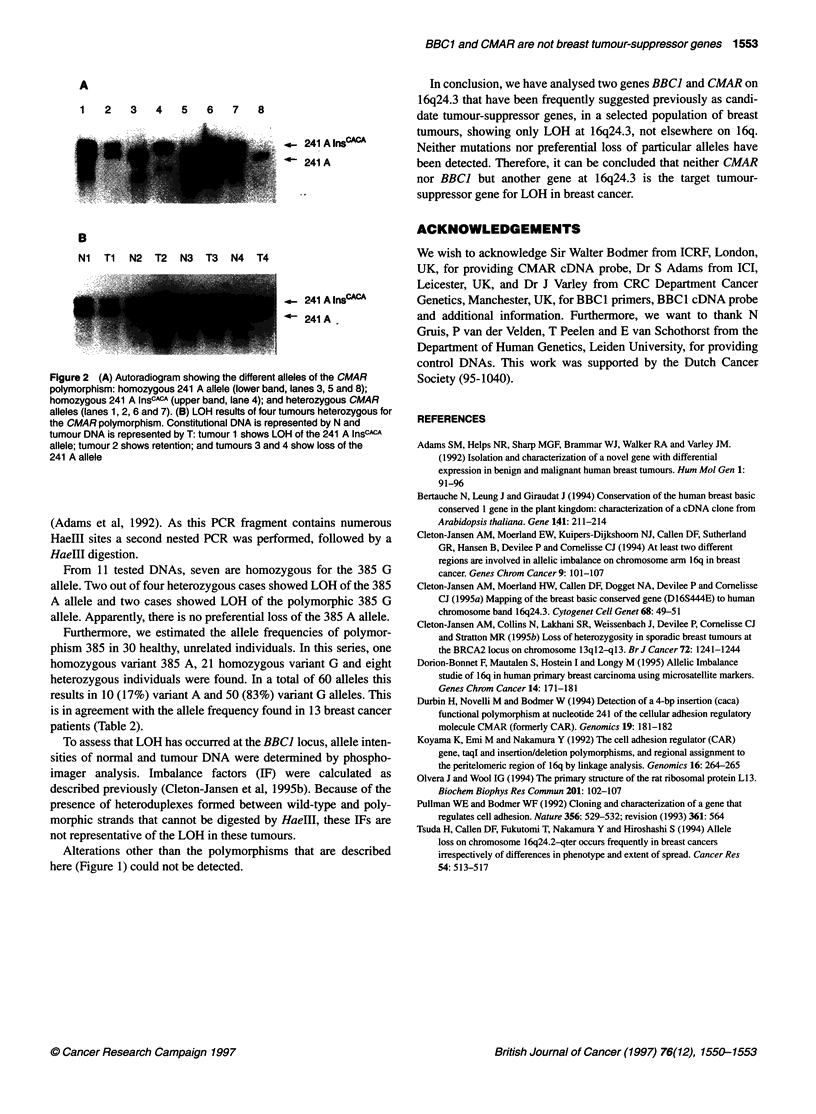# Exclusion of BBC1 and CMAR as candidate breast tumour-suppressor genes.

**DOI:** 10.1038/bjc.1997.594

**Published:** 1997

**Authors:** E. Moerland, M. H. Breuning, C. J. Cornelisse, A. M. Cleton-Jansen

**Affiliations:** Department of Pathology, Leiden University Medical Center, The Netherlands.

## Abstract

**Images:**


British Journal of Cancer (1997) 76(12), 1550–1553
© 1997 Cancer Research Campaign

### Exclusion of *BBC1* and *CMAR* as candidate breast tumour-suppressor genes

**E Moerland[1], MH Breuning[2], CJ Cornelisse[1] and AM Cleton-Jansen[1]**

Departments of [1]Pathology and [2]Human Genetics, Leiden University Medical Center, PO Box 9600, 2300 RC Leiden, The Netherlands

**Summary** Loss of heterozygosity (LOH) on chromosome arm 16q occurs in 48–65% of breast tumours. One small region of overlap is located at 16q24.3. Two genes located in this region, the cellular adhesion regulatory molecule (*CMAR*) and the breast basic conserved gene (*BBC1*), are plausible candidate tumour-suppressor genes. Mutational analysis of the retained copy of these genes has been performed by direct sequencing in a selected set of breast tumours that show LOH at 16q24.3 but not at other regions on chromosome arm 16q. In *CMAR* no other alterations than the previously described 4-bp insertion of *CACA* at nucleotide 241 could be detected, which was also present in constitutional DNA of the same patients. This polymorphism occurs homozygously in germline DNA of normal individuals and breast cancer patients. LOH analysis at this locus shows no preferential loss of a particular variant of the 241 polymorphism. In the *BBC1* gene, three different alterations were found, but only one resulted in an amino acid substitution. This is a known polymorphism, however, also appearing in germline DNA. The absence of tumour-specific mutations in *CMAR* and *BBC1* in this selected series of breast tumours implies that another gene at 16q24.3 must be the tumour-suppressor gene that is the target for LOH in breast cancer.

**Keywords:** breast cancer; tumour-suppressor gene; chromosome 16; breast basic conserved gene 1; cellular adhesion regulatory molecule

Loss of heterozygosity (LOH) on the long arm of chromosome 16 occurs frequently in breast cancer and other tumours. Percentages of LOH in breast cancer vary from 48% to 65% in different regions of 16q in different studies (Cleton-Jansen et al, 1994; Tsuda et al, 1994; Dorion-Bonnet et al, 1995). This suggests the presence of tumour-suppressor genes on this chromosome arm. One of two identified small regions of overlap is located at 16q24.3 between the markers APRT and D16S303 (Cleton-Jansen et al, 1994).

Two genes, the cellular adhesion regulatory molecule (*CMAR*) and the breast basic conserved gene (*BBC1*), located in this region are probable candidates for the gene targeted by LOH (Adams et al, 1992; Pullman and Bodmer, 1992; Cleton-Jansen et al, 1995*a*).

The *CMAR* gene product enhances binding of integrins to extracellular matrix components (Pullman and Bodmer, 1992). A 4-bp insertion of CACA at nucleotide 241 occurs in 38% of Caucasians and in 30% of Japanese (Koyama et al, 1992; Durbin et al, 1994). This 4-bp insertion probably results in an alternative start site for the gene (Durbin et al, 1994).

The *BBC1* gene was identified by differential screening of cDNA libraries of a primary breast carcinoma and of a benign fibroadenoma (Adams et al, 1992). Expression of this gene is higher in fibroadenomas than in carcinomas. The gene is expressed in a great variety of tissues and it is also highly conserved in other species (Bertauche et al, 1994). At the genetic level 85% homology is found with the gene coding for the rat ribosomal protein L13, at the protein level 97% homology is found (Olvera et al, 1994).

*Received 23 January 1997*
*Revised 29 May 1997*
*Accepted 3 June 1997*

*Correspondence to:* E Moerland, Department of Pathology, Leiden University Medical Center, Building 1, L1-Q, PO Box 9600, NL-2300 RC Leiden, The Netherlands

To investigate whether *CMAR* or *BBC1* is the tumour-suppressor gene on 16q24.3 targeted by LOH, we performed mutational analysis of the retained copy of these genes in breast tumours that show LOH at 16q24.3.

Moreover, we have addressed the question whether the polymorphisms in *CMAR* and *BBC1* can occur homozygously and if any of the variants of the polymorphisms is specifically deleted by LOH.

### MATERIALS AND METHODS

### Tumour specimen and control DNA

For this analysis DNA and RNA were isolated from a set of 16 breast tumours of the infiltrating ductal type, varying from stage I to stage IV and with age of onset between 28 and 82 years. All tumours were removed from patients attending the Leiden University Hospital. Thirteen cases showed LOH only at 16q24.3 and not at other regions of chromosome arm 16q. Three cases showed a complex LOH, i.e. loss at 16q24.3 and 16q22.1, and retention in between. One tumour had LOH at the whole 16q arm (Figure 1). This set was selected from a series of 230 sporadic breast tumours on which LOH analysis had been performed with 37 polymorphic markers on chromosome arm 16q (partly described in Cleton-Jansen et al, 1994).

For estimating the allele frequencies of the polymorphisms described here, control DNA isolated from healthy unrelated individuals from the Leiden region was used.

### Detection of the 241 A Ins$^{CACA}$ CMAR polymorphism

To detect the polymorphism polymerase chain reaction (PCR) reactions were carried out in 12 µl of reaction volumes of a mixture containing: 100 ng DNA; 0.2 mM dNTPs (optional 1 mC [$^{32}$P]dCTP); 6 pmol oligonucleotides; 0.06 U Super *Taq*; and 1 × Super *Taq* buffer (HT Biotechnology, Cambridge, UK). Reactions were performed in a thermocycler (MJ Research) under the following conditions: 1 min at 95°C; 1 min at 55°C; and 1.5 min at 72°C for 32 cycles. PCR products were separated on a 6% denaturing acrylamide gel and visualized by autoradiography.

Primers used for amplification were:

CMAR For 2: 5′-TCCAAACAGACCCCTGTTC-3′
CMAR Rev 3: 5′-AGCTCCTCTGCATTAGAGCTG-3′

The reverse primer CMAR Rev 3, located at the 3′ UTR, was designed to screen the complete coding sequence from *CMAR*. This was performed by sequencing the *CMAR* cosmid NL-87D4. This cosmid was obtained by screening a chromosome 16-specific library with *CMAR* cDNA probe, kindly provided by Sir Walter Bodmer.

Subsequently, a digestion with *Bgl*I for non-radioactive detection was performed. The 241 A Ins$^{CACA}$ destroys a *Bgl*I restriction site.

### Mutation analysis of CMAR

Non-$^{32}$P-labelled *CMAR* PCR fragments were screened for mutations after Easy Prep system purification (Pharmacia) by cycle sequencing (1 min at 95°C; 1 min at 55°C; and 1.5 min at 72°C for 32 cycles) with a cycle-sequencing kit (Amplicycle sequencing kit, Perkin Elmer) with nested primers:

CMAR For 1: 5′-ATGCCGATGCTTGCAC-3′
CMAR For 3: 5′-TAGTCACGCATGCAGTGTTGG-3′
CMAR Rev 2: 5′-GTATCTCAACAATTCTCAGAGCAG-3′

### Screening of the BBC1 gene

RNA was isolated from frozen breast tumour tissue using TRIZOL reagent (Gibco BRL). Reverse transcription was performed on 2 μg of total RNA, using an Oligo(dT) primer and 5 U of AMV reverse transcriptase (Boehringer Mannheim), in 20-μl reactions containing the recommended buffer, 5 U of RNAsin (Promega) and 1 mM dNTPs.

PCR reactions were performed on 1 μl of cDNA (1 min at 95°C; 1 min at 59°C; and 1.5 min at 72°C for 32 cycles).

Primers used for amplification of the *BBC1* coding sequence:

BBC For: 5′-TTTCCGCTCGGCTGTTTT-3′
BBC Rev: 5′-CGACTGATTCCAAGTCCCC-3′

Cycle sequencing (1 min at 95°C; 1 min at 60°C; and 1.5 min at 72°C for 32 cycles) was performed using the following primers:

BBC For nest: 5′-GCAGGAGCCGCAGGGCCGTAG-3′
BBC Rev nest: 5′-GTCCCCAGGAGGGCTTTATT-3′
BBC 10-9 (For): 5′-GGTACCACACGAAGGTGCGC-3′
(kindly provided by S Adams).

### Detection of the BBC1 polymorphism on genomic DNA

A first non-radioactive PCR was performed on 100 ng of DNA in a 50-μl volume (1 min at 95°C; 1 min at 55°C; and 1.5 min at 72°C for 12 cycles).

BBC 10–10 (For): 5′-CTCTGTTGGCCTCAGTCGCG-3′
BBC 620 (Rev): 5′-ACTCCTTCAGCCGCTG-3′ (both primers provided by S Adams).

A second, seminested PCR was carried out at the same conditions, for 20 cycles with 1-μl input from the first PCR and incorporation of $^{32}$P, in a reaction volume of 50 μl.

**Table 1** Frequency of the *CMAR* 241 A polymorphism in a normal control population and in breast cancer patients

| *CMAR* alleles | Control population | Breast cancer patients |
|---|---|---|
| Homozygous 241 A | 83 (72%) | 119 (69%) |
| Homozygous 241 A Ins$^{CACA}$ | 2 (1.8%) | 4 (2.3%) |
| Heterozygous | 29 (25%) | 49 (28%) |

BBC Eχ2 (For): 5′-GCAGGAGCCGCAGGGCCGTAG-3′
BBC 620 (Rev): 5′-GCAGGAGCCGCAGGGCCGTAG-3′

As the polymorphism creates a *Hae*III restriction site, a HaeIII digestion was performed resulting in a 24 bp shorter band on 6% denaturing polyacrylamide gel.

### RESULTS AND DISCUSSION

### Occurrence of the 241 A Ins$^{CACA}$ polymorphism in CMAR

The polymorphism results in a 4 bp larger PCR fragment, which is detected on a denaturing polyacrylamide gel. Allele frequencies on 228 chromosomes of unrelated Caucasions were 85% for allele 241 A (lower band) and 15% for the 241 A Ins$^{CACA}$ polymorphism. Observed homo- and heterozygosity rates were similar to the expected rates, i.e. 72% homozygous allele 241 A, 1.8% homozygous polymorphism Ins$^{CACA}$ and 26% heterozygous (Table 1). The occurrence of this polymorphism is similar to previously described frequencies of 30% (Koyama et al, 1992) and 38% (Durbin et al, 1994). However, homozygosity of 241 A Ins$^{CACA}$ has not been observed previously, probably because of low sample numbers. Figure 2A shows examples of homozygous allele 241 A, homozygous 241 A Ins$^{CACA}$ and heterozygous *CMAR* alleles as detected by PCR and autoradiography.

The detection of two normal individuals that are homozygous for the *CMAR* 241 A Ins$^{CACA}$ allele shows that absence of one of the variants of the *CMAR* polymorphism is not deleterious. This either implicates that the 241 A Ins$^{CACA}$ allele encodes a functional protein or that *CMAR* is not an indispensable gene.

### LOH at CMAR in breast tumours

Constitutional DNA of 172 breast cancer patients was used to determine the allele frequency of the *CMAR* 241 A Ins$^{CACA}$ polymorphism. On 344 chromosomes, the frequency was 83% for allele 241 A and 17% for the 241 A Ins$^{CACA}$ allele. Observed homo- and heterozygosity rates are similar to the frequencies observed in the normal control population as shown in Table 1.

A series of 168 breast tumours was tested for LOH at the *CMAR* locus using the 241 A Ins$^{CACA}$ polymorphism. Of the 38 informative cases 25 (66%) showed LOH. This high LOH rate is in concordance with previous results with other markers from this chromosomal region (Cleton-Jansen et al, 1994). If *CMAR* is involved in tumour suppression, and if one of the variants encodes a less active protein, one would expect preferential loss of one of the alleles. However, of these cases 13 showed LOH of the 241 A allele and 12 showed LOH of the 241 A Ins$^{CACA}$, indicating no preferential loss of allele 241 A.

Figure 2B shows examples of LOH of the 241 A (T3, T4) and the 241 A Ins$^{CACA}$ (T1) *CMAR* alleles.

© Cancer Research Campaign 1997 

**Figure 1** LOH results at 16q24.3 (LOH results on 23 markers proximal from 16q24.3 are summarized) and mutation analysis of *BBC1* and *CMAR* of 16 breast tumours. White, retention; black, loss of heterozygosity; grey, not informative; X, not tested or not interpretable; #, no sequence deviations from the GenBank sequence (GenBank accession no. *CMAR*: D14075; *BBC1*: S54769), CACA, insertion on nucleotide 241 A of *CMAR*; 144 G, 192 T, 385 G, polymorphisms in *BBC1*

### Mutation analysis of CMAR in breast tumours

Somatic mutation analysis for CMAR in breast cancer has been reported previously by Koyama et al (1992), who did not detect mutations. However, Koyama et al did not screen the complete coding sequence, 21 nucleotides are missing because one of the primers used is located in the coding sequence. As not all breast tumours show LOH at 16q and as this chromosome arm is suspected to contain at least two different tumour-suppressor genes, we have repeated this analysis on a more specific set of breast cancer cases, i.e. tumours with LOH only at 16q24.3, not on other regions of chromosome arm 16q (Figure 1). Figure 1 shows the LOH results obtained with markers on 16q24.3 and more proximal markers, which were described in Cleton-Jansen et al (1994).

The *CMAR* gene does not have introns and the complete sequence was amplified in one PCR reaction on genomic DNA from the tumours, using primers according to the submitted sequence (GenBank accession no. S54769) and the sequence from 3′UTR that was determined by sequencing a *CMAR*-positive cosmid, NL-87D4. Subsequent sequencing of these PCR products revealed no sequence deviations other than the already known polymorphism (Figure 1).

### Mutation analysis of BBC1 in breast tumours and polymorphisms in the BBC1 gene

From 13 breast tumours presented in Figure 1, RNA was available for analysis of the *BBC1* gene. RT-PCR fragments consisting of the complete coding sequence of *BBC1* were generated from this tumour cDNA and sequenced.

**Table 2** Polymorphisms in the *BBC1* gene in 13 breast tumours

| Base position | Polymorphism | Amino acid change | Sequence according to GenBank | Deviation from GenBank sequence |
|---|---|---|---|---|
| 144 | T → G | No change | 11 (85%) | 2 (15%) |
| 192 | C → T | No change | 11 (85%) | 2 (15%) |
| 385 | A → G | Thr → Ala | 2 (15%) | 11 (85%) |

Three different deviations from the GenBank sequence were found in this gene (Figure 1), two of which did not result in an amino acid change, 144 T → G and 192 C → T. One alteration, 385 A → G resulted in an amino acid substitution, but this is a known polymorphism (J Varley, personal communication) that appears also in germline DNA from the same patient. Table 1 shows the frequency of the polymorphisms found in this study.

Polymorphism 385 G, the only alteration that results in an amino acid change, occurs in 11 tumours whereas the 385 A allele, concordant with the GenBank sequence, is seen in only two cases. To determine whether this is a preferential loss of the 385 A allele, we examined constitutional DNA extracted from peripheral lymphocytes from the same patients. A two-step PCR was designed: the first PCR with a forward primer in intron 1 and the reverse primer in exon 2 to avoid amplification of pseudo genes

© Cancer Research Campaign 1997

**Figure 2** (**A**) Autoradiogram showing the different alleles of the *CMAR* polymorphism: homozygous 241 A allele (lower band, lanes 3, 5 and 8); homozygous 241 A Ins$^{CACA}$ (upper band, lane 4); and heterozygous *CMAR* alleles (lanes 1, 2, 6 and 7). (**B**) LOH results of four tumours heterozygous for the *CMAR* polymorphism. Constitutional DNA is represented by N and tumour DNA is represented by T: tumour 1 shows LOH of the 241 A Ins$^{CACA}$ allele; tumour 2 shows retention; and tumours 3 and 4 show loss of the 241 A allele

(Adams et al, 1992). As this PCR fragment contains numerous HaeIII sites a second nested PCR was performed, followed by a *Hae*III digestion.

From 11 tested DNAs, seven are homozygous for the 385 G allele. Two out of four heterozygous cases showed LOH of the 385 A allele and two cases showed LOH of the polymorphic 385 G allele. Apparently, there is no preferential loss of the 385 A allele.

Furthermore, we estimated the allele frequencies of polymorphism 385 in 30 healthy, unrelated individuals. In this series, one homozygous variant 385 A, 21 homozygous variant G and eight heterozygous individuals were found. In a total of 60 alleles this results in 10 (17%) variant A and 50 (83%) variant G alleles. This is in agreement with the allele frequency found in 13 breast cancer patients (Table 2).

To assess that LOH has occurred at the *BBC1* locus, allele intensities of normal and tumour DNA were determined by phosphoimager analysis. Imbalance factors (IF) were calculated as described previously (Cleton-Jansen et al, 1995b). Because of the presence of heteroduplexes formed between wild-type and polymorphic strands that cannot be digested by *Hae*III, these IFs are not representative of the LOH in these tumours.

Alterations other than the polymorphisms that are described here (Figure 1) could not be detected.

In conclusion, we have analysed two genes *BBC1* and *CMAR* on 16q24.3 that have been frequently suggested previously as candidate tumour-suppressor genes, in a selected population of breast tumours, showing only LOH at 16q24.3, not elsewhere on 16q. Neither mutations nor preferential loss of particular alleles have been detected. Therefore, it can be concluded that neither *CMAR* nor *BBC1* but another gene at 16q24.3 is the target tumour-suppressor gene for LOH in breast cancer.

### ACKNOWLEDGEMENTS

We wish to acknowledge Sir Walter Bodmer from ICRF, London, UK, for providing CMAR cDNA probe, Dr S Adams from ICI, Leicester, UK, and Dr J Varley from CRC Department Cancer Genetics, Manchester, UK, for BBC1 primers, BBC1 cDNA probe and additional information. Furthermore, we want to thank N Gruis, P van der Velden, T Peelen and E van Schothorst from the Department of Human Genetics, Leiden University, for providing control DNAs. This work was supported by the Dutch Cancer Society (95-1040).

### REFERENCES

Adams SM, Helps NR, Sharp MGF, Brammar WJ, Walker RA and Varley JM. (1992) Isolation and characterization of a novel gene with differential expression in benign and malignant human breast tumours. *Hum Mol Gen* **1**: 91–96

Bertauche N, Leung J and Giraudat J (1994) Conservation of the human breast basic conserved 1 gene in the plant kingdom: characterization of a cDNA clone from *Arabidopsis thaliana*. *Gene* **141**: 211–214

Cleton-Jansen AM, Moerland EW, Kuipers-Dijkshoorn NJ, Callen DF, Sutherland GR, Hansen B, Devilee P and Cornelisse CJ (1994) At least two different regions are involved in allelic imbalance on chromosome arm 16q in breast cancer. *Genes Chrom Cancer* **9**: 101–107

Cleton-Jansen AM, Moerland HW, Callen DF, Dogget NA, Devilee P and Cornelisse CJ (1995*a*) Mapping of the breast basic conserved gene (D16S444E) to human chromosome band 16q24.3. *Cytogenet Cell Genet* **68**: 49–51

Cleton-Jansen AM, Collins N, Lakhani SR, Weissenbach J, Devilee P, Cornelisse CJ and Stratton MR (1995*b*) Loss of heterozygosity in sporadic breast tumours at the BRCA2 locus on chromosome 13q12–q13. *Br J Cancer* **72**: 1241–1244

Dorion-Bonnet F, Mautalen S, Hostein I and Longy M (1995) Allelic Imbalance studie of 16q in human primary breast carcinoma using microsatellite markers. *Genes Chrom Cancer* **14**: 171–181

Durbin H, Novelli M and Bodmer W (1994) Detection of a 4-bp insertion (caca) functional polymorphism at nucleotide 241 of the cellular adhesion regulatory molecule CMAR (formerly CAR). *Genomics* **19**: 181–182

Koyama K, Emi M and Nakamura Y (1992) The cell adhesion regulator (CAR) gene, taqI and insertion/deletion polymorphisms, and regional assignment to the peritelomeric region of 16q by linkage analysis. *Genomics* **16**: 264–265

Olvera J and Wool IG (1994) The primary structure of the rat ribosomal protein L13. *Biochem Biophys Res Commun* **201**: 102–107

Pullman WE and Bodmer WF (1992) Cloning and characterization of a gene that regulates cell adhesion. *Nature* **356**: 529–532; revision (1993) **361**: 564

Tsuda H, Callen DF, Fukutomi T, Nakamura Y and Hiroshashi S (1994) Allele loss on chromosome 16q24.2–qter occurs frequently in breast cancers irrespectively of differences in phenotype and extent of spread. *Cancer Res* **54**: 513–517

© Cancer Research Campaign 1997